# Assessment of an Artificial Intelligence Tool for Estimating Left Ventricular Ejection Fraction in Echocardiograms from Apical and Parasternal Long-Axis Views

**DOI:** 10.3390/diagnostics14161719

**Published:** 2024-08-08

**Authors:** Roberto Vega, Cherise Kwok, Abhilash Rakkunedeth Hareendranathan, Arun Nagdev, Jacob L. Jaremko

**Affiliations:** 1 Exo Imaging, Santa Clara, CA 95054, USA; 2 Department of Radiology and Diagnostic Imaging, Faculty of Medicine and Dentistry, University of Alberta, Edmonton, AB T6G 2R3, Canada; ctkwok@ualberta.ca (C.K.); hareendr@ualberta.ca (A.R.H.); jjaremko@ualberta.ca (J.L.J.); 3 Alameda Health System, Highland General Hospital, University of California San Francisco, San Francisco, CA 94143, USA; arun@exo.inc

**Keywords:** left ventricular ejection fraction, echocardiogram, ultrasound imaging, machine learning, artificial intelligence

## Abstract

This work aims to evaluate the performance of a new artificial intelligence tool (ExoAI) to compute the left ventricular ejection fraction (LVEF) in echocardiograms of the apical and parasternal long axis (PLAX) views. We retrospectively gathered echocardiograms from 441 individual patients (70% male, age: 67.3 ± 15.3, weight: 87.7 ± 25.4, BMI: 29.5 ± 7.4) and computed the ejection fraction in each echocardiogram using the ExoAI algorithm. We compared its performance against the ejection fraction from the clinical report. ExoAI achieved a root mean squared error of 7.58% in A2C, 7.45% in A4C, and 7.29% in PLAX, and correlations of 0.79, 0.75, and 0.89, respectively. As for the detection of low EF values (EF < 50%), ExoAI achieved an accuracy of 83% in A2C, 80% in A4C, and 91% in PLAX. Our results suggest that ExoAI effectively estimates the LVEF and it is an effective tool for estimating abnormal ejection fraction values (EF < 50%). Importantly, the PLAX view allows for the estimation of the ejection fraction when it is not feasible to acquire apical views (e.g., in ICU settings where it is not possible to move the patient to obtain an apical scan).

## 1. Introduction

Left ventricular ejection fraction, which is the proportion of blood ejected during systole, is one of the most important measurements to evaluate cardiac function [1]. Echocardiography using ultrasound devices [2] is a preferred tool for computing the ejection fraction because of its widespread availability, its cost effectiveness, and its non-invasive nature [3]. Although its proper estimation is critical for diagnosis and clinical decision making, its computation is far from trivial. 

The traditional pipeline for computing the ejection fraction of the left ventricle from ultrasound involves the following: (1) the identification of the end-systole and end-diastole frames in two orthogonal apical views, (2) the tracing of the endocardial borders of the left ventricle at these frames, and (3) the use of a model-based calculation, such as the bi-plane method of disk summation [4]. In practice, this computation is a subjective process that depends on both the quality of the acquired images and the experience of the reader [1,5,6]. Obtaining high-quality images traditionally requires high-end ultrasound equipment and highly skilled operators [7]. 

In non-traditional imaging settings, like the emergency department, imaging is performed by physicians with different backgrounds and levels of experience with ultrasound imaging [8]. The growth of rapid bedside ultrasound imaging (commonly referred to as point-of-care ultrasound) has grown in part due to low-cost, portable handheld ultrasound machines that are used at bedside when comprehensive echocardiograms are not immediately available [9]. However, handheld devices typically have lower image quality compared with full-sized cart devices, adding more uncertainty to the computation of the ejection fraction [10,11].

Since the full pipeline for computing the EF is very time consuming, a commonly used method is a visual estimation of the ejection fraction, also known as ‘eyeballing’ [12,13]. This method is fast and allows for a real-time estimation of the cardiac function, but its efficiency depends heavily on the experience of the health provider [12,13,14]. Besides time constraints, image acquisition in point-of-care settings is challenging, since it is often not possible to move and reposition an acutely ill patient optimally. Therefore, it is not always possible to properly acquire the views typically used to compute ejection fraction (apical two-chamber and apical four-chamber views), forcing health providers to rely on easier-to-obtain cardiac views, such as the parasternal long axis [4,15].

To address these challenges, several studies have successfully assessed the feasibility of using artificial intelligence to automatically compute the ejection fraction of the left ventricle. Some methods use black-box models, which take as an input a sequence of ultrasound images and compute the ejection fraction without the need of computing the end-systole and end-diastole volumes [4,15]. Alternatively, a second set of methods use artificial intelligence to identify the borders of the left ventricle, automatically find the end-systole and end-diastole frames, and then use either Simpson’s method or the bi-plane summation-of-disks method to compute the ejection fraction [1,6,7,10,12,14,16,17,18]. Here, we assess the performance of an artificial intelligence algorithm that lies in this second set.

The use of AI is particularly important for point-of-care ultrasound. The objective of point-of-care ultrasound is to answer focused questions at bedside rather than to perform a comprehensive evaluation of an anatomical region [19]. Its use is rapidly expanding in fields like emergency medicine, critical care, anesthesiology, primary care, etc. [20]. However, ultrasonography is a user-dependent technology, and its proper interpretation requires adequate training [21]. Artificial intelligence is a technology that helps overcome some of the issues related to ultrasound imaging, including reducing user variability and the required training for interpreting the images. A proper evaluation of AI tools is critical for their success [5,22].

Since it is not always possible to obtain all the standard views (apical four-chamber and apical two-chamber views) in critically ill patients in the acute setting, it becomes important to evaluate the performance of artificial intelligence algorithms when only one of the views is available or when only a non-standard view like the parasternal long axis is possible. Here, we evaluate the performance of an algorithm that computes the left ventricular ejection fraction using the following views: apical four-chamber, apical two-chamber, and parasternal long axis.

### Goals of This Investigation

Our research objective was to assess the performance of a newly available artificial intelligence algorithm (ExoAI) that automatically computes the left ventricular ejection fraction (LVEF) in echocardiograms. Specifically, we compared the LVEF calculated by ExoAI from the A2C, A4C and PLAX views to each other and to the LVEF from the clinical report of the patients. This comparison was made in terms of the root mean squared error, linear regression analysis, Bland–Altman analysis, as well as accuracy, sensitivity, specificity and the positive predicted value in identifying an ejection fraction lower than 50%.

## 2. Materials and Methods

We retrospectively gathered data from 441 patients from 12 different imaging clinics in Edmonton, AB, Canada, who received an echocardiography exam between Jan-2022 and Mar-2023, and whose echocardiogram contained at least two full cardiac cycles. The mean age of the patients was 67.3 years (SD 15.3), their mean weight was 87.7 kg (SD 25.4), their mean body mass index was 29.5 (SD 7.4), and 70% were male (309/441). The patients generally presented for a calculation of left ventricular ejection fraction, but overall, there were diverse indications for scanning. Patients had atrial fibrillation (*n* = 76), pericardial effusion (*n* = 32), aortic stenosis (*n* = 31), pulmonary hypertension (*n* = 12), atrial septal defect (*n* = 3), valvular regurgitation (*n* = 24), pulmonary hypertension (*n* = 12), or cardiomyopathy (*n* = 5).

Out of the 441 patients, 92% (407/441) had an apical 4-chamber view, 69% (304/441) had an apical 2-chamber view, and 61% (269/441) had a parasternal long-axis view. The clinical report of each patient included the ejection fraction computed independently from A2C, A4C and bi-plane views. These values were used as reference values when comparing them against the ejection fraction computed by the artificial intelligence algorithm. Since the ejection fraction is not traditionally computed from the parasternal long-axis view and was therefore not included in the clinical report, a senior cardiac sonographer with 14 years of experience manually computed the ejection fraction from this view. The sonographer first identified the end-systole and end-diastole frames, measured the left ventricular internal dimension, and computed the ejection fraction using the Teicholz method.

Finally, we compared the performance of using the PLAX view to estimate the ejection fraction measured from the apical views. We used the ejection fraction in the report and compared its value against (1) the PLAX ejection fraction estimated by the senior cardiac sonographer and (2) the ejection fraction estimated by the AI algorithm.

### 2.1. Measurements

**Figure 1:**
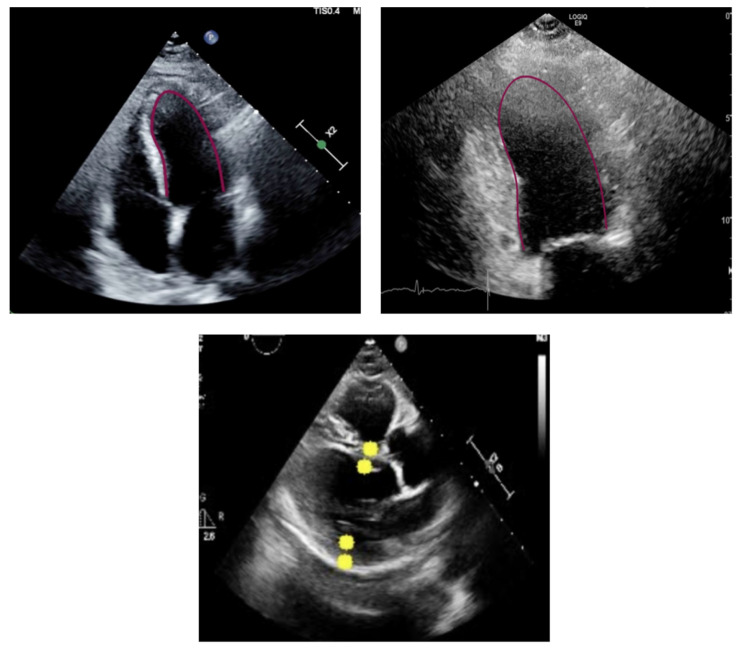
Examples of the predicted contour (purple lines) and landmarks (yellow dots) of the left ventricle in different views: A4C (**top left**), A2C (**top right**), and PLAX (**bottom**).

ExoAI is a proprietary software tool developed by Exo Inc. (Santa Clara, CA, USA, version 2.1.0). It has two different pipelines, one for apical views (A2C or A4C) and one for PLAX view. We input each of the recorded echocardiograms, one at the time, to the appropriate pipeline and recorded the computed ejection fractions. The output on apical views includes the tracing of the endocardium in apical views in end-diastole and end-systole frames and the resulting EF measurement. For the PLAX views, the output includes the left ventricle internal dimension (LVID), interventricular septum (IVS) thickness and posterior wall (PW) thickness and the resulting EF measurement, as depicted in Figure 1.

The artificial intelligence pipeline includes 4 different phases: (1) the identification of the contour of the left ventricle in the apical views or the corresponding landmarks in the parasternal long-axis view, (2) the identification of all the end-systole/end-diastole frames present in the echocardiogram, (3) an estimation of the left ventricular volumes using Simpson’s method for the apical views and the Teicholz method for the PLAX view, and (4) the computation of the ejection fraction based on the end-systolic and end-diastolic volumes. Since there are multiple cardiac cycles in each echocardiogram, the artificial intelligence system reports the median value of the ejection fraction, as well as its corresponding confidence intervals. 

These 4 steps are computed automatically, without the need of any human intervention other than providing the input echocardiogram and its corresponding view. As part of the contour/landmarks identification process, the artificial intelligence tool automatically detects frames where the predictions are suboptimal, and it eliminates those frames from the ejection fraction computation. This process occurs in the background. If the predictions have a high level of uncertainty, the system outputs “inconclusive results”.

### 2.2. Data Analysis

The results computed by the artificial intelligence algorithm were compared with the values outlined in the clinical report for the apical views and with the value estimated by the senior cardiac sonographer for the PLAX view. Additionally, we compared the PLAX ejection fraction computed by both the senior cardiac sonographer and the artificial intelligence algorithm against the apical values in the clinical report. All the comparisons are summarized by the root mean squared error, intraclass correlation coefficient, Pearson’s correlation coefficient, as well as bias, variance and interquartile range of the error between the predicted and reported values.

We also evaluated the performance of the artificial intelligence tool for identifying cases where the clinically reported ejection fraction was lower than 50%. Such cases were considered as true positive. We report the total number of false positives, false negatives, true positives and true negatives. Additionally, we compute the accuracy, specificity, sensitivity and positive predictive value.

### 2.3. Ethics Approval

Our retrospective study was approved by the health research ethics boards of each of the participating centers.

## 3. Results

Figure 1 exemplifies the output produced by the artificial intelligence algorithm. For the case of the apical views, the algorithm predicts the contour of the left ventricle. For the PLAX view, it predicts the location of four landmarks: two located in the outer walls and two located in the inner walls. Note how the algorithm can identify the contour even in lower-quality images where the walls of the left ventricle are not fully visible.

**Figure 2:**
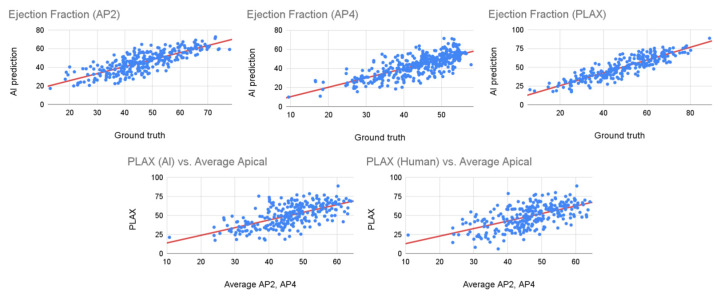
Linear regression analysis for the AI predictions and reference values.

Figure 2 (top) shows scatterplots of the values used as ground truth (clinical report for apical views and measurements by a senior cardiac sonographer for PLAX) against the values predicted by the artificial intelligence algorithm. Note that a linear regression properly follows the trend in the data. Table 1 shows numerical values that confirm this graphical interpretation. Pearson’s correlation between the predicted values and the reference values is above 0.7 for the three views, with PLAX predictions having a correlation of 0.89. Note that although the bias in the PLAX view is slightly higher than the ones in apical views, its variance is slightly smaller. Regarding the bias in the PLAX view, the variance values are (1.43 ± 7.16) while the values are (−0.88 ± 7.54) and (−0.15 ± 7.45) for the apical two chambers and apical four chambers, respectively. Finally, the interquartile range shows that most of the time, the absolute error is below 6.5% for all the views.

Figure 2 (bottom) compares the values estimated using the PLAX view from both the human sonographer and the AI algorithm against the values reported from the apical views. When the clinical report included the values for both apical two and four chambers, we used their average as the reference. If only one view was available, we used that reported value as a reference. Note that although the values are linearly correlated, the spread is higher for both the cardiac sonographer and the AI.

The last two columns of Table 1 provide a numerical interpretation of Figure 2. Note how the correlation between the PLAX EF and apical EF is 0.59 for the cardiac sonographer and 0.62 for the AI algorithm. Similarly, the bias is 2.66 and 4.09, respectively. Although these are promising values, they are clearly higher than those from the previous experiment. This result is expected because, in apical views, the end-systolic and end-diastolic volumes are estimated from the entire 2D contour, while in the PLAX views, it is estimated from a single 1D measurement, so we expect some error when estimating apical EF from the PLAX view.

**Figure 3:**
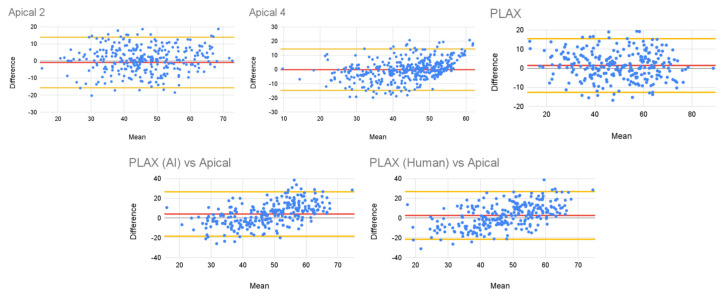
Bland–Altman plot for the AI predictions and reference values. The red lines represent the mean value, while the yellow lines represent the confidence intervals.

The Bland–Altman plots in Figure 3 (top) show the error in the measurement as a function of the expected value of the ejection fraction. For all the views, we can observe that the error is relatively constant across the full set of ejection fraction values, and most of the errors lie within the confidence intervals (−15.7, 13.91) for the apical two-chamber view, (−14.76, 14.46) for the apical four-chamber view, and (−12.61, 15.47) for the PLAX view.

Figure 3 (bottom) shows a similar analysis for the estimation of the apical EF from the PLAX view. Note how there is a small positive slope in the trend in the predictions. In other words, the EF is slightly overestimated from the PLAX view when the apical EF is greater than 50% and slightly underestimated for cases when the apical EF is lower than 50%. This trend occurs in both measurements taken by the cardiac sonographer and those taken by the AI algorithm.

Finally, Table 2 shows the performance of the algorithm in identifying cases where the reported ejection fraction was lower than 50%. The accuracy for the apical views was above 80%, with a sensitivity of 75% and 73% for the apical two-chamber and apical four-chamber views, respectively. Similarly, the specificity was 88% and 83% and the positive predictive value was 86% and 87%. For the PLAX view, all the metrics were above or equaled 90%, with an accuracy of 91%, sensitivity of 90%, specificity of 92% and positive predictive value of 93%. These results are also expected since it is easier to predict the diameter of the left ventricle, which is a 1D measurement, than to predict the contour of the left ventricle, which is a 2D measurement.

Importantly, note that although the correlation between the PLAX EF and apical EF was around 0.6, the accuracy in detecting cases with an EF lower than 50% is 69% for both the cardiac sonographer and the AI algorithm. Interestingly, the positive predictive value is 86% for the cardiac sonographer and 87% for the AI algorithm. These results indicates that whenever the PLAX view predicts a low ejection fraction, there is high probability that this is indeed the case.

### Limitations

This study was performed using retrospective data in patients well enough to attend 1 of 12 outpatient clinics for echocardiography. It was limited to 441 individual patients and was not focused on patients with focal myocardial pathology (e.g., apical myocardial infarcts). Also, because of clinical reasons out of the control of this study related to factors such as body habitus and the logistics of clinic workflow, not all the patients had the three analyzed views (A2C, A4C, and PLAX) available. However, it was unlikely that there was a systematic reason related to the LVEF that prevented some views from being obtained in specific patients. We did not have an external gold standard such as cardiac MRI or contrast ultrasound for our LVEF. Finally, for the estimating the performance of the artificial intelligence tool in the PLAX view, we had to use an average of the ejection fraction reported from the apical views, since the ejection fraction is not typically computed from PLAX echocardiograms.

## 4. Discussion

Assessing the left ventricular ejection fraction (LVEF) is critical for evaluating cardiac function [1], but obtaining high-quality images through traditional echocardiographic methods is time consuming and subjective. It depends on the operator’s experience, skill, equipment, and image quality [1], which often poses challenges in emergency settings where optimal imaging conditions and skilled echocardiographers are not always available [1,5,7]. Similarly, there is evidence of a higher inter- and intra-rater variability when assessing the left ventricular function in patients with a concave chest wall [23]. 

While point-of-care ultrasound devices improve accessibility, its production of lower-quality images compared to traditional methods adds uncertainty to LVEF estimations [9,10]. Visual estimation methods such as ‘eyeballing,’ are fast but highly dependent on the clinician’s expertise [12,13]. As such, AI offers promising solutions to these limitations where AI algorithms automate the identification of ventricular borders and key frames to compute LVEF more reliably [7,14]. Our results demonstrated that an AI algorithm, ExoAI, can accurately predict clinically low LVEF views in different views. The difference between using the ExoAI artificial intelligence tool and the manual computation of the ejection fraction is small, even in cases where image quality is sub-optimal—i.e., the walls of the left ventricle are not fully visible during the entire exam. 

In many real clinical scenarios, it is difficult to obtain an echocardiogram from the apical view to estimate the ejection fraction [4,15,24]. This is a common problem in emergency medicine scenarios, where it is often not possible to place the patient in the optimal position for imaging. For such cases, it might be necessary to estimate the ejection fraction from an alternative view, such as PLAX. Previous studies have shown that while both views are effective in predicting LVEF, PLAX has a potentially stronger association [25,26]. It is also considered more useful for estimating LVEF in critically ill patients compared to the apical four-chamber view [27]. The current study aligns with these findings, demonstrating that ExoAI achieved high accuracy in low clinically reported LVEF, with PLAX having the highest sensitivity (92%) and specificity (90%). 

Since the PLAX view does not directly visualize the cardiac apex, we expect the PLAX view to overestimate the LVEF in cases of focal apical pathology such as an apical myocardial infarction, which were not heavily represented in our data. Other studies have shown that combining AP4 and PLAX views can provide a more comprehensive assessment of LVEF [4]. For example, A4C excels in visualizing the LV apex and offers more consistent LV volume measurements, while PLAX is more reliable for assessing left atrium and aortic root dimensions [28]. Consequently, future studies on patients with suspected or known focal apical pathology are necessary, and relying solely on PLAX should be approached with caution when apical pathology is clinically suspected. 

A key advantage of using artificial intelligence is that it computes the ejection fraction from each cardiac cycle. It can then determine the most likely value of the ejection fraction after analyzing the information of the entire echocardiogram. It also allows the algorithm to compute confidence intervals of the prediction, allowing it to provide a more reliable estimation. Computing the ejection fraction following this procedure manually would be infeasible due to the amount of time and effort required. Additionally, utilizing POCUS can be quickly adopted for practical use. Studies have shown that POCUS-naive novices in emergency room settings can reliably acquire high-quality views of the PLAX and A4C views [15]. This capability is further supported by evidence showing that emergency physicians can quickly and accurately estimate global LVEF using PLAX and A4C views [29,30]. This shows that POCUS paired with artificial intelligence could be an accessible and easily adopted tool to improve cardiac assessment in emergency room settings. 

## Figures and Tables

**Table 1:** Evaluation of the ejection fraction computed by the artificial intelligence tool when compared with the value outlined in the clinical report.

	Apical 2	Apical 4	PLAX	AP vs. PLAX (Expert)	AP vs. PLAX (AI)
RMSE	7.58	7.45	7.29	12.57	12.16
Pearson’s R	0.79	0.75	0.89	0.59	0.62
Bias	−0.88	−0.15	1.43	2.66	4.09
SD	7.54	7.45	7.16	12.31	11.47
Min	−18.77	−19.81	−16.80	−31.1	−25.90
IQR: 25%	−6.25	−4.25	−3.40	−6.56	−3.44
IQR: 50%	−0.98	−0.01	1.14	3.31	4.34
IQR: 75%	3.84	3.69	5.84	11.54	10.81
Max	20.31	20.76	19.36	38.75	38.47

**Table 2:** Performance of the artificial intelligence tool in identifying cases where the reported ejection fraction was lower than 50%. The number of people with EF < 50% is the sum of true positives (TP) and false negatives (FN). The number of people with EF ≥ 50% is the sum of true negatives (TN) and false positives (FP).

	Apical 2	Apical 4	PLAX	AP vs. PLAX (Expert)	AP vs. PLAX (AI)
TP	172	237	124	119	117
TN	81	89	120	66	68
FP	27	33	10	19	17
FN	24	48	14	64	66
Accuracy	83%	80%	91%	69%	69%
Sensitivity	88%	83%	90%	65%	64%
Specificity	75%	73%	92%	78%	80%
PPV	86%	88%	93%	86%	87%

## Data Availability

The datasets presented in this article are not readily available because of privacy reasons and because of the conditions set on the data sharing agreement with the medical institutions where the data were collected. Requests to access the datasets should be directed to Jacob L. Jaremko: jjaremko@ualberta.ca.
